# Hospital and Institutional News

**Published:** 1916-09-30

**Authors:** 


### HOSPITAL AND INSTITUTIONAL NEWS.

### ALEXANDRA DAY DISTRIBUTION.

The Distribution Committee of the "Alexandra Day" Fund met on September 26 at the Mansion House, and distributed about £23,500 among various hospitals and charities of London. It is stated that awards amounting to £8,095 were made on the personal recommendation of Queen Alexandra herself; and that in the distribution of the rest of the money due attention has been paid to the wishes of the mayors and local committees in the various districts. The list of Queen Alexandra's awards is published; that of the charities benefiting by the committee's deliberations will be made public in due course. The two largest of Her Majesty's awards are £500 apiece to the Church Army and the British Home and Hospital for Incurables, Streatham. We notice that one institution, as far away as Belfast, has received £50. Without labouring this point, it is surely obvious that there are grave objections to this system of double control. Queen Alexandra can have, in the nature of things, no direct knowledge of the four or five score institutions on her personal list; it would be a far more satisfactory plan in every way if the whole distribution were effected by the committee, aided by one or more representatives from the King Edward VII. Hospital Fund.

### THE DAVID HOLLIN NURSING HOME.

A remarkable conditional legacy has been bequeathed to the Staffordshire General Infirmary under the will of the late Mr. David Hollin, who, subject to his wife's interest, has left £12,000 to the Staffordshire General Infirmary for the erection of a nurses' home, to be called after him. The testator provided that if this gift had been anticipated, the £12,000 should be paid to the Bishop of Lichfield for the building of a church at North End to be known as St. David's. Such a gift is a good instance of the position in which the nursing profession is held to-day, and of the recognition of the need for providing them with separate and properly equipped quarters. One result of this may well be the raising of the standard of wardmaids' quarters, which are still too often mean and cramped, and the one exception to the hospital standard of ventilation and general amenity. Now that nurses' homes can attract definite legacies the day is nearer when the public will equally realise the desirability of giving to the wardmaids and servants separate bedrooms and fully equipped quarters of their own. It is the business of the hospitals to educate the public in this matter also.

### A SPECIAL MEDICAL EXHIBITION.

The permanent Wellcome Historical Medical Museum in Wigmore Street is already one of the familiar institutions of London. Just at present it is especially worth a visit, because it contains a valuable loan collection of materials illustrating the folk-lore of London, lent by Mr. E. Lovett, of Croydon, a member of the Council of the Folk-lore Society. As far as possible Mr. Lovett has confined his collection to objects of British origin, but London is so cosmopolitan that foreign influences on native customs are inevitable, and are easily traceable in some of the exhibits. The collection is divided into six sections. In the first are many kinds of cures for diseases, in the way of amulets, charms, and lucky mascots generally. A potato carried as a cure for rheumatism is a superstition which is still widely diffused, and similar magical cures for nightmare, cramp, small-pox, kidney diseases, bronchitis, diarrhœa, and many other diseases are also shown: "rheumatic rings," as many people know, are still sold in large numbers in London, and are even worn by persons of education. In the second section are charms consisting of horns, tusks, claws, and similar animal remains. The third section is devoted to charms against lightning, and the remaining sections are different varieties of mascots for good fortune of various kinds.

### THE THERAPEUTIC VALUE OF EXAMPLE.

To assist the Leeds Hospital Handicrafts Fund a sale of work was lately held, at which Lieutenant-Colonel Littlewood, administrator of the 2nd Northern General Hospital, presided. In the course of a speech he remarked on the extraordinary manner in which men who were collected together as suffering from the same disease improved in health and spirits by this association. A blind man, who was so miserable that he contemplated suicide, was restored to cheerfulness on his removal from a hospital where the other patients had normal eyesight to an institution for patients sightless like himself. Similarly, disabled men also improved when placed among others suffering from a similar injury. One man learnt from another, watched the progress of others, and gained knowledge and hopefulness from seeing others struggle with difficulties of the same nature as his own. Perhaps the only exception to this rule is in the case of mental sufferers and asylum patients, of whom it can be said with less confidence that they benefit from being surrounded by persons equally afflicted in minds. The moral of these observations seems to be that sufficient institutions must be provided to give each blind or disabled soldier a chance of benefiting from association to the full.

### A SUGGESTIVE APPEAL.

Just as indirect influences affect the lives of most of us far more than direct ones, so the art of appeal-writing largely consists in interesting people so that they may feel a wish to support that which interests them rather than in asking them straight out to give. This principle has been worked out with considerable care and skill in an agreeable pamphlet issued on behalf of the Glasgow Royal Infirmary, entitled "The Mansion House: Glasgow's Greatest Guest House: Its Purpose and Problems." The senior physician, the senior surgeon, the consulting medical electrician, and, presumably, the secretary, have combined to produce a pamphlet which will make the chief departments of the hospital interesting to the lay reader, and to infect him with the life and energy that centre in the great institution. Type, paper, and illustrations have been judiciously chosen, and, as will be guessed from the title of the pamphlet, an element of surprise is introduced. Only the expert would detect " an appeal " in its grey covers; forms for donations, bequests, or annual subscriptions are conspicuous by their absence. In particular, we do not think the word " appeal " occurs. There is no direct request for money. The fact that the hospital has only voluntary gifts to depend on is mentioned in the course of the description, but that is all. The cost of the pamphlet itself has been given by " four friends of the infirmary," and the little note which records this on the last page will not be wasted. Titles are so hard to choose that it seems ungenerous to criticise this one. The matter and manner of presentation show care and skill. We hope to receive news of the results achieved by it.

### A HOSPITAL WAR SAVINGS ASSOCIATION.

Guy's Hospital has set a useful example in forming a War Savings Association. It has only been in existence since August 1, but can already boast 221 members. Sir Cooper Perry is the chairman, Mr. Curry (clerk to the hospital) is treasurer, and Miss Huggett, secretary. The members, who are drawn from every department of the hospital, from the consulting staff to the wardmaids, have subscribed over £100. A mass meeting to explain the purposes of the Association was held as a preliminary, and collectors for every department were appointed by the Committee, notices to that effect being posted everywhere so that all might know where to find their collector. Two hundred and three subscriptions were taken the first week (6d. being the unit), and in the fifth the number of new ones had risen to 931, while eighty-three certificates have already been allotted. The certificates with the earliest dates are given to those who first complete their payments; if several finish on the same day preference is given to those who began first, while if several began and end on the same day the certificates are given in alphabetical order. The example of Guy's Hospital might well be followed by other institutions up and down the country.

### NAVAL HOSPITAL ARRANGEMENTS.

The Medical Department of the Royal Navy, according to information which has already been published in the daily press, has elaborated a scheme whereby the casualties in the next Grand Fleet action, if ever there is one, shall receive the promptest and best possible attention, whereever they may happen to be landed, and however numerous they may be. The scheme which has been worked out has been rendered feasible only by the willing co-operation of the Army Medical Service, of civil authorities, and of voluntary hospitals and aid organisations all along the Eastern coasts of these islands. A large number of military hospitals have promised that, whenever a great naval battle takes place, numbers of convalescent or semi-convalescent soldier patients shall be rapidly evacuated further inland, and their beds used for the treatment of naval casualties. Civil hospitals have arranged to increase the number of beds in their wards, and to equip temporary wards at a moment's notice in out-patient departments and other buildings. Civic authorities, private owners, and the St. John Ambulance Association have combined to devise a system of motor-transport, whereby the wounded in their cots can be debarked with the minimum of trouble. Nor is it only in the large ports that these arrangements have been thought out. Wherever a trawler or other " mosquito " craft can put in, the system is in working order, waiting for the call; let us hope it will never need to be put to the test.

### MILITARY BEDS IN COTTAGE HOSPITALS.

The desirability of devoting beds in cottage hospitals to wounded soldiers is one that it is time to consider now that the general plans for the hospital accommodation of wounded men are in full working order. Wishing to show their patriotism, the managers of various cottage hospitals offered a ward or some beds, which were often conditionally accepted. In several instances the military have found no occasion to use them, and the question therefore arises as to whether, in such cases, the truly patriotic duty of cottage hospital managers is not to reconsider their decision. A case in point is that of the Fleetwood Cottage Hospital. In this institution a ward of ten beds was reserved for wounded men. Not, we learn from the annual statement, until the year had almost passed were any soldiers sent for treatment. Then a small number arrived. In spite of this slight use the ward continues to be kept in constant readiness, and in the opinion of the honorary secretary, the Rev. A. Bailey, this reservation of beds must continue. It may be said in defence of this view that owing to military service the normal number of cottage hospital patients tends to decline. But the problem does not end here. The large hospitals have alarmingly big waiting lists. We therefore ask whether the cottage hospitals, whose size makes them less suitable for military patients, would not be doing a more patriotic work if they could be employed to take some of the less serious waiting cases from the lists of big hospitals nearest at hand. The difficulties in the way of such co-ordination are obvious, but they should not prove overwhelming. The matter at all events demands active attention.

### VICTIMISING A CHILDREN'S HOSPITAL.

The directors of the Royal Hospital for Sick Children, Glasgow, have issued through Mr. Robert F. Barclay, the honorary secretary, a warning against impostors who, he says, " are making a regular system " of getting subscriptions from the public. Apparently mostly women, they are ingenious enough to employ some modification of the hospital's proper name, and find, it seems, the possession of an annual report accepted as sufficient evidence that they are authorised to collect funds on its behalf. So old and simple a form of fraud should be sufficiently understood by this time, but many people are appallingly slow to realise their responsibility, and would rather get rid of a collector by giving a small sum than by examining their credentials. Such persons are as morally reprehensible as those whom they allow to impose upon their laziness, but the trouble is that the injury really falls not upon them but on the hospital. If the sum annually amassed by these bogus collectors could be estimated it would be found that the hospital had been defrauded of a large sum. Anyone who has allowed himself to be imposed upon cannot as an honest man do less than send the sum which he gave to the wrong person once more to the proper source; for he has, in fact, incurred by his fault a debt to the hospital which it is his duty to repay.

### SURGICAL DRESSINGS FROM THE U.S.A.

Mrs. Mary Hatch Willard, the chairman of that valuable organisation the National Surgical Dressings Committee, which was formed in the United States, a month after the outbreak of war, to supply the Allied Forces with surgical material, has come to London to arrange for even closer co-operation in supplying requisites as special needs arise. Local branch committees were formed under the parent organisation in every State of the Union, and over eight million surgical dressings have already been supplied. The committees are all self-supporting; the funds of the movement are stated to be ample, and there is a constant flow of gifts and money coming in. Patterns of desired articles are supplied by the Allied Governments, and are distributed throughout the United States to those who work for the committee. Everything is done free, and many American families have sent in linen sheets to be made into dressings, sometimes sacrificing in this way family treasures of long storing. Everything is done on requisition, so that there is no waste and no doubling.

### WAR CHARITIES REGISTRATION.

The Registration of War Charities proceeds apace, and one more charity—namely, the Albert Day scheme—has been added to those to be administered by the Charity Commissioners. This fund has been collected by a Mr. Jos. Clarkson, on behalf of the Belgian Canal Boat Fund, which organisation, however, disassociated itself some time ago from Mr. Clarkson's method of raising money. *Truth* is asking why the "Everyman" Fund, organised for Belgian relief and reconstruction work by Dr. Sarolea, has not been added to the list. There is under this organisation a sum of £20,000 earmarked for "repatriation" purposes after the war, and it is obvious that nobody could object to the Charity Commissioners taking care of the money. But the Commissioners are by no means idle, and up to the last report 120 war charities in the County of London have registered. It is felt in many quarters, as pointed out by Lord Plymouth in a letter to the *Times*, that the audit regulations are rather irksome. It is difficult enough in these times to get an annual audit, let alone one every three months. But, as another correspondent of the same journal suggests, it is more important that an audit of some war charities should be held early rather than often, for then undesirable features of finance could be rectified before they are carried too far.

### AN IMPORTANT PROSECUTION UNDER THE PUBLIC HEALTH ACT.

Much interest has been aroused in the North of England by the successful prosecution of a confectioner at St. Helens, by the medical officer of health there. The offence charged was that of exposing for sale chocolate cream which was unfit for human consumption; and the novel point in the case was that the unfitness alleged by the prosecution was contamination of the sweetmeat by flies. The shop swarmed with flies, and thirty of them were crawling on the chocolate cream in the window, where the sun had melted it and had actually caused four flies to be imbedded. That the stuff was unfit for consumption is abundantly clear, and the action of the M.O.H. in instituting measures for condemning it and for prosecuting the owner was quite justified. At the same time, as some of the Northern newspapers have urged, it was not proved that the abundance of flies in the shop was due to any default of the confectioner, whose neighbours may conceivably have been more to blame for leaving refuse uncovered than he was for failing to prevent the flies bred therein from getting at the chocolate. It may be hoped that the public conscience will be stirred up sufficiently to make tradesmen more alert in the interests of the public health.

### A NEW HOSPITAL FOR FACE INJURIES.

The Joint Committee of the British Red Cross Society and Order of St. John have provided a new hospital of thirty beds at Kennington, to be opened on October 1, for discharged soldiers suffering from facial wounds and deformities capable of being remedied by plastic surgery. There are, of course, several Army hospitals where men still on the strength are sent for treatment of this sort: the King George V. Hospital is perhaps the most notable instance in London where this form of plastic surgery is to be seen at its best. There ought not to be any need for a similar institution for discharged soldiers, for the reason that the Army ought not to discharge them until everything that is possible has been done for them. Still, the fact that the Joint Committee have thought it worth while to start this "Maxillo-facial" hospital may be taken as evidence that the need exists. There is, however, one point in connection with the announcement about which some misgiving is permissible. The impression is given in the daily press that this hospital will be staffed by dental surgeons, and particularly that American dental surgeons have much to teach us in the treatment of these mutilating wounds. It will be distinctly detrimental to the wounded if the treatment of these cases comes to be regarded as outside the province of ordinary plastic surgery, and the exclusive domain of the dental surgeon. The latter's aid is indispensable, but the recent attempts by a section of the dental profession to get the reversion, as a right, of all cases of facial disfigurement due to wounds are much to be deprecated; and it is to be trusted that the Joint Committee will see that first-rate surgeons are in charge at Kennington, with an efficient staff of dentists as collaborators.

### SKEGNESS COTTAGE HOSPITAL.

We much regret having to record the death of the chairman of the Skegness Cottage Hospital, Lieutenant S. Coetmore Jones, agent to the Earl of Scarborough, the chief landowner in this district. Soon after the present war broke out, Mr. Coetmore Jones, in his forty-fifth year, without waiting for a commission, enlisted, and, by careful attention to his duties, was appointed lieutenant. He proved himself a valiant soldier and died a noble death, serving his country. Two of his ancestors held important commands at Waterloo. As chairman of the executive and one of the trustees of the Cottage Hospital at Skegness, Mr. Jones did splendid work. His *forte* was architecture, and his knowledge of plans and ability for finance made him an expert in this direction. He took a great interest in the recent extension of the hospital buildings, and will be greatly missed He leaves a widow and two young daughters. The Parish Church was crowded at the memorial service on September 16.

### MAJORITY VOTES ON WORKMEN'S CONTRIBUTIONS.

At an adjourned meeting of the governors of Pontypool and District Hospital the representatives of the workmen were asked to consent to contribute one penny per week extra for six months on the ground that, with the employers' quota of 25 per cent. on these contributions, a sum would be obtained sufficient to liquidate the existing overdraft. After some discussion a majority of about two-thirds was obtained in favour of the additional payment. Whether the minority will fall in line with this decision remains to be seen. Their objections seem to be based partly on the ground that weekly contributions to various objects are already numerous, and therefore a burden to a man with family responsibilities, and partly in the belief that the overdraft could be paid by the sale of investments. The secretary, Mr. Tom Morgan, was careful to explain that the present was not the time to sell, since stocks had depreciated in value. This being so, an increased contribution is the only solution; and it is to be hoped that all the men, once realising this, may join with their fellows to save the financial situation.

### RETIREMENT OF A VETERAN HOSPITAL CHAIRMAN.

On the occasion of the meeting of the governors of the Royal Portsmouth, Portsea, and Gosport Hospital held on Friday, September 22, the Rev. W. Hawksley, Vicar of All Saints, Portsea, who has held the position of chairman for two successive years, declined again to stand for election in consequence of his acceptance of the living of Michelmersh. This retirement marks the close of a remarkable and prolonged record of hospital service. Having become a member of the committee of management nearly twenty-six years ago on undertaking the office of honorary chaplain to the institution, he has occupied a seat upon that committee ever since, except for an interim period following his resignation of the chaplaincy in 1905. Restored to membership of the committee later on he has since filled the positions of vice-chairman and of chairman of the committee of management, and of vice-chairman and chairman of the governing body, and has twice in the last-named capacity presided at the annual meeting of subscribers. It was in consequence of the efforts and action of Mr. Hawksley that eligibility for membership of the committee of management was secured for women, and that thus the institution became a pioneer in welcoming women upon hospital governing boards.

### THE B.M.A. AND THE COMMISSION OF INVESTIGATION.

The existence of a Commission of Investigation appointed by the Faculty of Insurance to consider the position of National Insurance has led to a correspondence between the British Medical Association and the National Health Insurance Joint Committee, in which the Association asked whether the Commission of Investigation were intended to include in its survey matters connected with the medical service under the Insurance Acts. The Insurance Commissioners replied that the Commission of Investigation had no official standing, and that any questions affecting the interests of panel doctors were advisedly excluded from review by the Departmental Committee on Approved Society Finance and Administration appointed in January last. The British Medical Association, therefore, is free to ignore the present inquiry, and perhaps the chief value of the correspondence, so far as the public is concerned, is to emphasise the unofficial character of the Commission of Investigation, which has apparently been formed in the interests of certain approved societies. With the dispersal of the profession on war service abroad, and, indeed, at home, no time could be more inopportune for an official inquiry into the working of medical benefits under the Insurance Acts. The time will come for that. But it is not immediate nor likely to be.

### TASMANIAN HOSPITAL PENSION SCHEME.

The Hobart General Hospital, which the chairman of the hospital board describes as practically a Government institution, appears to be unable to grant pensions to retiring employees, however long and faithfully they have worked in the cause of the sick. To meet in some way the claims of members of this deserving class the board has prepared a scheme of gratuities and retiring allowances which it is thought will come within what Government will sanction. The scheme, which is good as far as it goes, is based on the system of public bodies in allowing so much sick leave in a given period with full pay and cash allowance for emoluments. The hospital leave for this purpose is one month in every year, so if a worker is so fortunate as to serve a number of years without much sick leave there will be a tidy cash sum to draw on retirement. There is a provision that should an employee die in the service his widow, family, or legal representative can draw what the employee would have been entitled to if he had retired at the same date that he died. The latter feature is generous and useful, but the former is a poor substitute for a pension.

### WHEN QUACKS FALL OUT.

A subtle compliment has been paid, it appears, to that exceedingly valuable compilation, issued by the British Medical Association, known as "Secret Remedies," by an unqualified medicine-monger who was lately hauled before the Vacation Court. This man, whose name is Dart, trades up and down the country as a cheap-jack in a waggonette, selling "medicines." He leads off with a lecture on patent medicines, in which he quotes "Secret Remedies" as his authority for the statement that the ingredients of a bottle of "Phosferine," (the much-advertised nostrum of another firm) cost one halfpenny. He then goes on to extol the virtues of his own "Phos-tonic," though he does not, apparently, disclose what the cost price of it is. He then sells pamphlets for a shilling, with a bottle of "Phos-tonic" thrown in. On one occasion he sold this preparation in a bottle which had once contained "Phosferine," and for this he was prosecuted by the incensed proprietors of that patent medicine. The judge said he was not prepared to make any order on the evidence before him, so the quarrel will be transferred to the ordinary Courts after the vacation is over. This use of "Secret Remedies" by an unqualified practitioner to discredit the product of a rival firm is new to us, and is highly ingenious.

### LIGHTENING THE LOAD.

With the object of diminishing the calls upon the time of medical practitioners undertaking the domiciliary treatment of insured persons suffering from tuberculosis, the Local Government Board has been well advised to abolish certain regulations made some four years ago, which required such practitioners to keep a continuous record of the clinical history of the illness of the patient and particulars of the treatment given to the patient, and to submit the card or sheet containing the record periodically to the consulting officer. The Order which rescinds these provisions contains, however, new regulations requiring practitioners to prepare and transmit to the consulting officers, at least once in every three months, a report in regard to each patient under their care, and to make arrangements for each patient to be examined by the consulting officer at least once a year. The new regulations do not come into force until January 1, 1917, and until that time medical practitioners carrying out the domiciliary treatment of insured persons suffering from tuberculosis must continue to submit the continuous record of the clinical history of the illness of the patient and particulars of the treatment to the consulting officer as heretofore.

### MILK DELIVERIES.

It is an old custom, not yet even moribund, for the dairyman to make morning and afternoon deliveries of milk. Originally the later delivery was of milk of a later milking, and the fiction survives that it is so. As a fact, farmers nowadays only too often keep the morning milk (under the best conditions they have available) until the afternoon, sending it and the afternoon milk to town together by a night train. The consumer is fortunate if the milk is only thus stored and mixed, and if no worse evil befall it by the way. Hospital boards of management and other large consumers have the control of the details of their milk supply in their own hands if they choose to avail themselves of their opportunities when entering into contracts. It is much to be regretted that so often they are content to ignore such details, and to rely with a blind faith upon legislation and the efforts of the public health authorities.

### THE RED CROSS AND BRITISH PRISONERS: THE NEW COMMITTEE.

The latest tribute to the respect felt for the Joint War Committee of the British Red Cross Society and the Order of St. John of Jerusalem has been the invitation of the War Office to it to take over all matters concerning the welfare of both combatant and civilian British prisoners of war, including those interned in neutral countries. A special committee, presided over by Sir Starr Jameson, Bart., C.B., has been formed to co-ordinate the efforts of various associations and private persons for the dispatch of comforts to British prisoners. Members appointed by the Joint Committee, the War Prisoners Help Committee, and a representative of the Indian Soldiers Fund will be included in the new body. It numbers seven, including the chairman, and by centralising control should much improve the organisation. The Red Cross Prisoners of War Committee and the Prisoners of War Help Committee now lapse and are superseded by the new committee.

### HOT BATHS FOR SOLDIERS.

For a very long time past the Emergency Voluntary Aid Committee of the Empress Club has been occupied with the excellent work of providing hot baths for our soldiers, both in field hospitals and in "reserve billets" for those just back from the trenches. It is true that the Army Medical Service does a great deal in this way, and that large numbers of bathing establishments are provided for our soldiers by the Army authorities; but this is certainly one of those comforts of which the men can hardly have too much, so that all voluntary help in the matter is useful. Lately the Navy has thrown itself into the scheme as a practical way of showing its admiration of the sister Service; without any canvassing on the part of the Empress Club, the crews of twenty-seven warships have provided funds for 140 baths and twenty-eight hot-water heaters, to be sent to battalions at the Front. The scheme is known as "Tubs for Tommies," and is a purely voluntary effort started by ladies of the Empress Club, Dover Street. Over 2,100 portable metal baths, with the necessary stoves and hot-water boilers, have been distributed already, and the club is continuing the provision of these welcome gifts as funds permit.

### NEWSPAPER READING IN MENTAL HOSPITALS.

In an interesting report on the Mental Hospital, Down, Dr. J. M. Nolan has some interesting things to say about the influence of the "war idea" on insanity, and about the effect of regulating the supply of books to the insane. After noting that a decline in the number of admissions has continued since the war started, a fact on which he laid some stress in his previous report, Dr. Nolan remarks that the "war idea" has had but little effect on the minds of his patients, and attributes this, in part at least, to the limits set on the free circulation of illustrated and unillustrated newspapers. To make up for a curtailment in this respect an additional supply of volumes has been placed in the day rooms. In other mental hospitals where the "war idea" is apparent it would be interesting to know whether or not the supply of newspapers is regulated. These facts show that the newspaper is as potent within the walls of an asylum as without.

### THE VALUE OF TUBERCULOSIS COLONIES.

The success of the colony system in the treatment of pulmonary tuberculosis is already an acknowledged fact. At the recent annual congress of the Incorporated Sanitary Association of Scotland, held in Edinburgh, an interesting lecture was delivered by County Councillor George Fraser, of the Middle Ward of Lanarkshire, upon graded labour for the cure of consumption as practised at Hairmyres. Mere vegetation at a sanatorium is of comparatively little value in the treatment of phthisis. The patient must be educated and occupied at the same time, not only that his mind may not become vacant, but also in order to secure the full physiological effect that is known to accrue from graduated exercises when properly carried out. It was pointed out that in the Hairmyres scheme the children would be kept entirely separate from the adults, and that the former would have their own workshops and gardens. Farming, market gardening, and forestry were introduced into the scheme, and such special branches as pig-rearing, seed-bed and nursery cultivation, in addition to saw-milling, would also be included. Under wise and skilful supervision there is no reason why graded labour, intelligently prescribed and followed out, should not play a yet greater part in sanatorium treatment than it does at the present time.

### THE HEALTH OF GUERNSEY.

The Channel Islands, which as part of the old Duchy of Normandy sometimes claim that England belongs to them, not they to England, are for all practical purposes independent States, which make their own laws and manage their own affairs. Broadly speaking, public health administration there is conducted on similar lines to those familiar in Great Britain, though there are minor differences. The annual report of the medical officer of health, Dr. H. D. Bishop, reveals tendencies which closely parallel the experiences of English districts of similar partly urban, partly agricultural type. There was in 1915 a very serious fall in the birth-rate below that for 1914 itself, the lowest on record: the number of births was 784, whereas the average for the previous ten years was 1,001. The death-rate, too, was above the average, as it was in most parts of England; it was 14.8 per thousand, compared with 11.8 in 1914, a serious difference. Happily the island was entirely free from typhoid and the paratyphoid fevers during the year, a fortunate circumstance in view of the military incursions from infected districts in France. The infantile death-rate of 144 per thousand was far too high, especially for an island with such a climate as that of Guernsey. This rate, and the average rate for the last few years, are a disgrace to the States of Guernsey. The medical officer of health uses some emphatic language, which is fully justified, and it is high time that concerted action was taken to end this reproach: in one parish the infantile mortality rate is actually 227 per 1,000!

### THIS WEEK'S DRUG MARKET.

The downward tendency in the values of some of the synthetic drugs continues, and salicylic acid and salicylate of soda are distinctly lower again; prices, however, are still too high to tempt purchasers. Phenazone is also much lower, and aspirin still tends downwards. On the other hand, phenacetin continues extremely scarce, and very high prices have to be paid for any small lots available; and the same applies to barbitone. The advanced price of camphor is firmly maintained, and peppermint oil and menthol are both dearer. Makers of potassium bromide continue busy, and there seems to be little likelihood of any further decline in price. There has been no further drop in the price of cocaine, but it is not improbable that the price will recede somewhat. Atropine is hardly obtainable except in small quantities. Senega root is much dearer. At the recent public sale of drugs in Mincing Lane lower prices were accepted for rhubarb, honey, and ergot of rye; senna-leaves, Cape aloes, and cardamoms also tended lower, but the high price of nux vomica was well maintained.

### TO OUR READERS.

**Contributions are specially invited from any of our readers to these columns. They should deal with topical subjects and news. They must be authenticated for the information of the Editor only. The minimum payment if published is 5s. There is no hard-and-fast rule as to space, but notes of about twenty lines in length are preferred.**

